# Sustainable synthesis of bakuchiol-mediated gold nanoparticles for drug delivery against bacterial strains and tumor microenvironments, and its *in silico* target proteins identification

**DOI:** 10.3389/fmolb.2024.1469107

**Published:** 2024-09-25

**Authors:** Pooja Mishra, Tabrez Faruqui, Sheeba Khanam, Mohd Khubaib, Irfan Ahmad, Mohd Saeed, Salman Khan

**Affiliations:** ^1^ Department of Biosciences, Integral University, Lucknow, Uttar Pradesh, India; ^2^ Department of Clinical Laboratory Sciences, College of Applied Medical Science, King Khalid University, Abha, Saudi Arabia; ^3^ Department of Biology, College of Sciences, University of Hail, Hail, Saudi Arabia

**Keywords:** bakuchiol, nanoparticles, green synthesis, anticancer, apoptosis

## Abstract

**Introduction:**

The sustained synthesis of gold nanoparticles (GNPs) has gained significant attention in biomedical applications. In this study, we explored the antibacterial and anticancer potential of bakuchiol-mediated gold nanoparticles (Bak-GNPs). Bakuchiol, a natural compound found in *Psoralea corylifolia* seeds, serves as both a reducing and stabilizing agent for green synthesis of GNPs. Our objectives include network analysis, molecular docking, synthesis of GNPs, characterization, and antipathogenic and anticancer efficacy of Bak-GNPs against lung and liver cancers.

**Methods:**

Protein-protein interaction networks were analyzed to identify effective protein targets for bakuchiol in lung and liver cancers. A molecular docking study was performed to validate the efficacy of the target protein against lung and liver cancer. Furthermore, Bak-GNPs were synthesized using bakuchiol and characterized by various techniques such as UV-visible spectroscopy, dynamic light scattering (DLS), zeta potential transmission electron microscopy (TEM), and Fourier-transform infrared (FTIR) spectroscopy, and their potential against pathogens and lung and liver cancers.

**Results:**

GNAI3 emerged as the most promising target, with a binding energy of −7.5 kcal/mol compared to PTGER3’s −6.9 kcal/mol, different characterization techniques revealed the successful synthesis of Bak-GNPs. Bak-GNPs exhibited potent antibacterial activity against both Gram-positive and Gram-negative bacteria, as confirmed by minimum inhibitory concentration (MIC) values. Bak-GNPs demonstrated significant anticancer effects on A549 (lung cancer) and HepG2 (liver cancer) cells, with IC_50_ values of 11.19 μg/mL and 6.6 μg/mL, respectively. Induction of apoptosis and inhibition of cell proliferation were observed in both the cell lines. The increased production of reactive oxygen species (ROS) contributes to its anticancer effects.

**Discussion:**

This study highlights promising biomedical applications of bakuchiol-mediated GNPs. This green synthesis approach using bakuchiol provides a sustainable method for producing nanoparticles with enhanced biological activities. Further exploration of the pharmacological properties and mechanisms of Bak-GNPs is required to optimize their therapeutic efficacy for clinical use.

## Graphical Abstract

**Figure:**
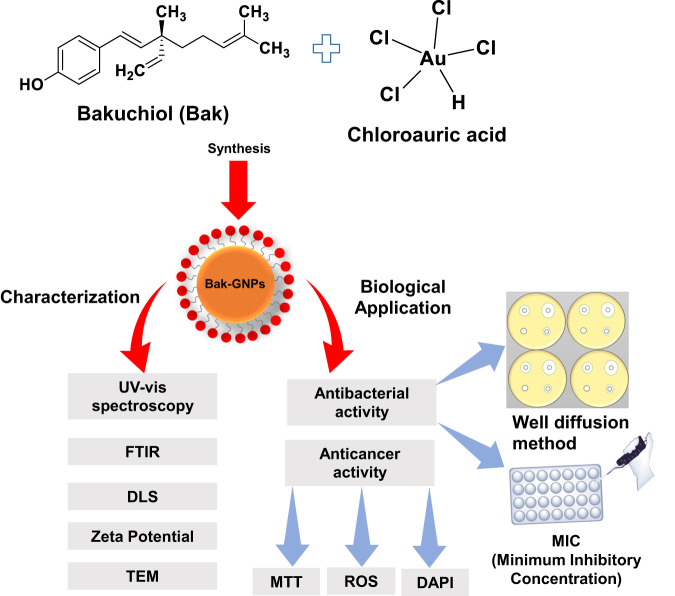
Graphical representation of the biogenic synthesis of gold nanoparticles.

## 1 Introduction

Cancer is a medical condition characterized by abnormal proliferation of cells or tissues. Worldwide, the primary challenge is the high death rate associated with cancer, primarily owing to the lack of effective treatments, particularly for malignant forms of the disease. Lung cancer is the most prevalent form of cancer in both men and women residing in developed countries. Furthermore, it holds the top spot as the leading cause of cancer-related fatalities on a global scale, accounting for 18.4% of all cancer deaths (Debela et al., 2021). At the time of diagnosis, approximately 70% of patients find themselves in the advanced stages of the disease, with only 15% surviving 5 years post-diagnosis (Blandin Knight et al., 2017). Several therapeutic approaches are commonly employed to treat lung cancer, including surgical intervention, radiotherapy, radiosurgery, chemotherapy, and immunotherapy. The selection of the most appropriate treatment depends on the patient’s functional assessment, disease stage, and histological type (Purandare and Rangarajan, 2015).

Green synthesis of nanoparticles, is environment-friendly and represents the drive towards chemical processes that minimize hazardous substances, lower energy consumption, and reduce harmful byproducts (Noah and Ndangili, 2022). This approach emphasizes economic viability, social responsibility, and ecological safety. In the context of green chemistry, green synthesis integrates nanotechnology into environmental sustainability. The eco-friendly production of GNPs combines these principles, leveraging the unique properties of these nanoscale gold particles for various industrial applications, while adhering to sustainable practices (Freitas de Freitas et al., 2018).

The use of bioactive compounds in the green synthesis of GNPs offers an innovative and environment-friendly method for creating nanomaterials by leveraging natural processes. This sustainable approach provides a safer alternative to traditional methods, which often involve hazardous chemicals (Venkatas et al., 2022; Cassani et al., 2023). Bioactive compounds found in various organisms, such as plants, fungi, and bacteria, include flavonoids, phenolics, terpenoids, and proteins. These compounds possess inherent biological activities and can interact at the molecular level, serving as reducing and stabilizing agents during the synthesis of GNPs. In addition, they may act as capping agents to prevent nanoparticle aggregation, thereby enhancing the efficacy and sustainability of the process (Saw et al., 2019).

Bakuchiol, a member of the meroterpenoid class, originates from the seeds and leaves of *Psoralea corylifolia*. It has a history of use in traditional medicine and is recognized for its antibacterial (Katsura et al., 2001), anticancer (Chen et al., 2010), antioxidant (Adhikari et al., 2003), and antiaging properties. Bakuchiol has recently emerged as a formidable defender against organ damage, offering protection against bone loss and delay in osteoporosis by exerting estrogen-like effects (Xin et al., 2019). As an analog of retinol, bakuchiol acts as an anti-aging compound through retinol-like regulation of gene expression (Chaudhuri and Bojanowski, 2014). Furthermore, Bakuchiol demonstrated potent cytotoxic effects in human gastric cancer cells (SGC-7901), exhibiting dose- and time-dependent effects (Lin et al., 2016).

## 2 Materials and methods

### 2.1 Material

Gold chloride (HAuCl_4_) was obtained from Sigma-Aldrich (St. Louis, MO, United States). Bakuchiol was procured from Cayman Chemical Company. DCFH-DA (2′,7′-dichlorodihydrofluorescein diacetate), and DAPI (4′,6-diamidino-2-phenylindole) were obtained from Sigma (St. Louis, MO, United States). Fetal bovine serum (FBS), Dulbecco’s modified Eagle’s medium (DMEM), 3-(4, 5-dimethylthiazol-2-yl)-2, 5-diphenyl tetrazolium bromide (MTT), Müeller Hinton (MH) broth, agar, and tryptone soy broth (TSB) were obtained from HiMedia (Mumbai, India). All other solvents and chemicals used were of analytical grade and were purchased from HiMedia Limited (Mumbai, Maharashtra, India).

### 2.2 Methods

#### 2.2.1 *In silico* study

##### 2.2.1.1 Protein-protein interactions of five chosen proteins

The present study comprehensively examined the proteins that contribute to cancer progression. Based on a literature survey, we identified five surface receptors as the most promising targets of bakuchiol for the treatment of lung and liver tumor microenvironments. A search tool for the retrieval of interacting genes that integrates both known and predicted PPIs was applied to predict the functional interactions of proteins. To identify the network of the most abundantly expressed genes, the top five genes with the highest level of upregulation were analyzed separately using the STRING database (http://string-db.org/) (Szklarczyk et al., 2021). Active interaction sources, including text mining, experiments, databases, and co-expression, as well as species limited to *“Homo sapiens”* with an interaction score > 0.71, were applied to construct the PPI networks. Subsequently, protein-protein interaction (PPI) networks derived from the STRING database were visualized and further analyzed using Cytoscape software, version 3.10, to determine the closeness centrality with the highest confidence score (Shannon et al., 2003).

##### 2.2.1.2 Protein and ligand retrieval, and docking study of closed interacting targets

All the target proteins listed in Supplementary Table 5, were obtained from the PDB database (https://www.rcsb.org/) in the form of their three-dimensional docking structures (PDB IDs: 3X23, 5TH6, 2HAZ, 3CFW, 1POZ, 7D85, 3VRQ, 4QSY, 7LEM, 1XDT, 4LOP, 7ECC, 4G2F, 2WO3, 3CZU, 2ODE, 6M9T, 6CRK, 7YK7, 2BCJ, 7S1C, 1UYL, 3T92, 8SWJ, and 6Q9L). The protein structure enhancement tools KoBaMIN and 3^Drefine^ were used to improve structural quality. Subsequently, each structure was subjected to energy minimization in a vacuum using GROMACS 2022.1 to eliminate any steric interference and further enhance the protein structure (Rodrigues et al., 2012; Bhattacharya et al., 2016). Ultimately, the protein structure was saved in the PDB format for future reference. To identify the most suitable target protein(s) for bakuchiol, we conducted molecular docking analysis of the top proteins ranked according to their confidence scores. In this study, we used *AutoDock Vina 4.1*, a widely used molecular docking software package (Trott and Olson, 2010). To conduct further investigation, the pose with the strongest binding affinity among the nine poses was selected.

##### 2.2.1.3 Gene expression evaluation

The TNMplot tool is well-suited for investigating discrepancies in gene expression within metastatic, normal, and malignant tissues (https://www.tnmplot.com/). The TNMplot is a comprehensive resource that includes 56,938 high-quality, multilayered samples, encompassing RNA-seq data from TCGA and GeneChip data from GEO. These samples were further categorized into 3,691 normal samples, 9,886 tumor samples, and 453 metastatic samples, making them a valuable database for researchers in the field of cancer genomics (Bartha and Győrffy, 2021). In this study, we analyed and compared the expression levels in healthy and malignant tissues. This tool was used to evaluate and assess the expression of RDX, ERBB3, EFNA5, GNAI3, PTGER3, and ATM in the normal, malignant, and metastatic tissues.

### 2.3 *In vitro* study

#### 2.3.1 Synthesis and characterization of surface-modified GNPs

For the synthesis of bakuchiol-functionalized gold nanoparticles (Bak-GNPs), we prepared reaction mixtures of 3 mL each. These mixtures consisted of 1 mM [HAuCl_4_] dissolved in 50 mM phosphate buffer and 330 μg/mL freshly prepared bakuchiol. The reaction mixture was then incubated at 40°C for 24 h. Notably, the color of the reaction mixture changed from white to red during incubation. To validate the synthesis of nanoparticles, we subjected the reaction mixture to UV-Vis spectroscopic analysis, followed by filtration through a 2 µm syringe filter. The resulting solution was stored at 4°C until further use (Soliman et al., 2020).

BioSpectrometer (Eppendorf AG, Germany.) can measure and record UV/Vis spectral ranges, as well as measure individual wavelengths from 200 nm to 830 nm. FTIR (Fourier-transform infrared) spectroscopy of gold nanoparticles is a technique used to study the interaction between infrared radiation and the surface of gold nanoparticles. This provides valuable information about the chemical composition and functional groups present on the nanoparticle surface. FTIR spectra for GNPs were obtained using a Perkin-Elmer Spectrum FTIR system (PerkinElmer Inc., Waltham, MA, United States) in the range of 650–4,000 cm^−1^ (Alshammari et al., 2021).

In the TEM studies, freshly synthesized gold nanoparticles were prepared by placing a drop of the nanoparticle solution onto carbon-coated copper grids and allowing the water to evaporate. Subsequent examinations were conducted using a Tecnai G2 Spirit transmission electron microscope with a BioTwin lens setup (Hillsboro, OR, United States). The microscope was operated at an acceleration voltage of 80 kV. Dynamic light scattering (DLS) was employed to determine the hydrodynamic radius of the synthesized nanoparticles, providing precise information about their submicron size. Zetasizer Nano-ZS (ZEN3600 Malvern Instrument Ltd., Malvern, United Kingdom) was used to assess nanoparticle stability and surface charge (Alshammari et al., 2021).

#### 2.3.2 Assessment of antibacterial activity

##### 2.3.2.1 Bacteria and growth conditions

Bacterial cultures of *Bacillus subtilis* (MTCC 441), *Escherichia coli* (ATCC 25923), *Klebsiella pneumoniae* (ATCC 13883), and *Staphylococcus aureus* (ATCC 25923) were obtained from the National Chemical Laboratory in India. Each bacterial strain was cultivated in Luria-Bertani (LB) broth at 37°C for 18 h. Before performing antibacterial tests, the optical density of each culture was adjusted to a McFarland standard of 0.5, equivalent to a concentration of 1.5 × 10^8^ CFU/mL, using LB broth (Soliman et al., 2020).

##### 2.3.2.2 Agar well diffusion method

The antimicrobial activity of the biosynthesized Bak-GNPs was assessed using the well diffusion technique against pathogenic bacteria, including *Bacillus subtilis* (MTCC 441), *Escherichia coli* (ATCC 25923), *Klebsiella pneumoniae* (ATCC 13883), and *Staphylococcus aureus* (ATCC 25923). Bacterial suspensions were evenly spread on the surface of Mueller–Hinton (MH) agar plates, and then wells were created into which Bak-GNPs, pure bakuchiol (Bak), and a negative control (PBS) were introduced. This experimental procedure was repeated thrice, and the agar plates were incubated overnight at 37°C. The diameters of the inhibition zones were then measured. In a subsequent experiment, it was observed that both pure bakuchiol (Bak) and Bak-GNPs diffused into agar, effectively suppressing bacterial growth (Alshahrani et al., 2021).

##### 2.3.2.3 MIC (minimum inhibitory concentration)

The minimum inhibitory concentrations (MICs) of the synthesized Bak-GNPs and pure bakuchiol against various bacterial strains were determined using the Eloff broth microdilution method. Samples of Bak-GNPs and pure bakuchiol were serially diluted to concentrations ranging from 0.78 to 50 μg/mL in 96-well microtiter plates containing LB broth medium. The bacterial strains under investigation were cultured in LB broth overnight, and their turbidity was adjusted to match the McFarland standard of 0.5, corresponding to a concentration of 1.5 × 10^8^ CFU/mL. The MICs represent the lowest concentrations of Bak-GNPs that completely inhibited bacterial growth following a 20 h incubation at 37°C (Eloff, 1998).

#### 2.3.3 Evaluation of cytotoxic and anticancer activities of Bak-GNPs

##### 2.3.3.1 Cell culture

Normal human primary osteoblasts, human lung cancer cells (A549), and liver cancer cells (HepG2) were obtained from the National Center for Cell Science (NCCS) in Pune, India. Both cell lines were grown as monolayers, with human primary osteoblasts cultured in McCoy’s medium, and A549 and HepG2 cells in DMEM. The culture medium was supplemented with 10% fetal bovine serum (FBS) and 1% actinomycin. The cell lines were subcultured regularly and maintained in a humidified incubator at 37°C. Routine checks were conducted to ensure the quality and purity of cell lines throughout the experimental period. Any deviations from the standard culture conditions have been documented and promptly addressed (Al Saqr et al., 2021).

##### 2.3.3.2 Assessment of cytotoxicity

The cytotoxic effect of Bak-GNPs on human lung (A549) and liver (HepG2) cancer cells was assessed using the MTT cytotoxicity colorimetric assay (Soliman et al., 2020). Initially, cells were seeded at a concentration of 1 × 10^4^ cells/well in a 96-well plate and incubated for 24 h at 37°C. Subsequently, the cells were exposed to GNPs in triplicate at concentrations ranging from 3.125–50 μg/mL for 48 h. Following this, MTT reagent (5 mg/mL) was added to each well. The plates were further incubated for an additional 4 h, and the formazan crystals that formed were dissolved by adding 150 µL DMSO to each well. Optical densities (OD) were measured at 570 nm with a reference filter of 655 nm using a Microplate Reader (BIORAD-680) to assess cell viability. The results were reported as the percentage of viable cells relative to untreated control cells (Mishra et al., 2023).

##### 2.3.3.3 Analysis of cytomorphological changes in (A549) and HepG2 cells

Pretreated GNPs (IC_50_) were added to (A549) and HepG2 cells and incubated at 37°C in 5% CO_2_. Gross changes in cell morphology were observed 24 h after incubation using an inverted phase-contrast microscope (Nikon ECLIPSE Ti-S, Tokyo, Japan).

##### 2.3.3.4 Detection of nuclear condensation

DAPI (4′,6-diamidino-2-phenylindole), a fluorescent dye used to mark cell nuclei, was used to assess the potential for apoptosis in A549 and HepG2 cells that had been exposed to GNPs at concentrations causing 50% inhibition of cell growth (IC_50_). Images of stained cells were acquired using a fluorescence microscope equipped with inverted optics (Nikon ECLIPSE Ti-S, Japan) (Tarnowski et al., 1993).

##### 2.3.3.5 Evaluation of intracellular reactive oxygen species (ROS) production

To identify the production of reactive oxygen species (ROS) in A549 and HepG2 cells treated with Bak-GNPs, the non-fluorescent compound DCFH-DA was used. DCFH-DA (2′–7′ dichlorofluorescin diacetate) can permeate cells and is retained within the intracellular environment after being cleaved by intracellular esterases. After encountering ROS-induced oxidation, the DCFH molecule transforms into the intensely fluorescent 2′,7′-dichlorofluorescein (DCF). Briefly, A549 and HepG2 cells (10,000 cells/well) were cultured in 24-well plates and incubated for 24 h at 37°C. The cells were exposed to IC_50_ concentrations of Bak-GNPs for 24 h. Subsequently, cells were treated with 10 μM DCFH-DA for 30 min at 37°C. Any residual DCFH-DA was removed by washing, and fluorescence microscopy (Nikon ECLIPSE Ti-S, Japan) was used to capture the images (Al-Rashed et al., 2021).

## 3 Results and discussions

### 3.1 Protein-protein interaction analysis

Information derived from protein-protein interaction (PPI) networks is indispensable for understanding cellular functions and biological processes. Numerous studies have demonstrated that proteins in close proximity to one another in a constructed PPI network frequently exhibit shared characteristics (Lee et al., 2004; Wurmbach et al., 2007; Wu et al., 2009). It can be deduced from numerous investigations that proteins within the subnetwork linking genes are likely to exhibit similar biological functions (Ideker and Sharan, 2008; Miryala et al., 2018). The present study used the STRING database, a widely recognized online repository of protein interactions, to construct a PPI network that incorporated both direct (physical) and indirect (functional) associations (Table 1). To determine which nodes in a network are essential, topological characteristics are crucial. The primary goals of topological property analysis include biological network analysis, pharmacological activity mechanisms, and protein identification. The PPI network in the Cytoscape tool was used to determine the topological aspects of the network using a Network Analyzer (Supplementary Figure 1). Network Analyzer using the Simple Interaction Format (.sif) in Cytoscape. The top five weighted genes (CD44, EPHA3, EFGR, PTGER3, and TP53) are shown in Table 1. One technique for identifying objects that can efficiently send data across a network is closeness centrality (CC). An excellent way to find objects that can transport data across a network is through closeness centrality (CC). A node’s proximity centrality determines the average distance between nodes. Our selection of the top five nearby genes for each protein was based on the criterion of high closeness scores, which indicates greater proximity to one another than to any other node in the network (Supplementary Figure 1). Network Analyzer using the Simple Interaction Format (.sif) in Cytoscape. The top five weighted genes (CD44, EPHA3, EFGR, PTGER3, and TP53).

**TABLE 1:** STRING network statistics.

S. No.	Protein/Gene	No. of nodes	No. of edges	Average node degree	Average local clustering coefficient	PPI enrichment p-value
1	CD44	21	108	10.3	0.75	1.11e-16
2	EPHA3	21	176	16.8	0.91	1.06e-16
3	EFGR	21	122	11.6	0.78	1.0e-16
4	PTGER3	21	164	15.6	0.91	1.0e-16
5	TP53	21	96	9.14	0.76	5.29e-07

### 3.2 Docking study of closed interacting targets

Molecular docking was performed to identify the best bakuchiol target. To validate the 25 shortlisted targets based on closeness centrality, the optimal target proteins were identified using *AutoDock Vina 4.1*. There are nine distinct docked postures for protein-ligand complexes produced by the versatile molecular docking tool *AutoDock Vina* (Trott and Olson, 2010). The initial posture with the highest binding affinity for each protein was selected from nine stances. After the docking process was completed, the best target proteins were selected based on their binding affinities. A more thorough revision docking procedure was performed for the best suited proteins. To determine the protein that would work best for bakuchiol in targeting lung cancer, a molecular docking study was conducted. Based on the docking data, GNAI3 had the highest binding energy of −7.5 kcal/mol. In contrast, PTGER3 had the second-highest binding energy of −6.9 kcal/mol, indicating that GNAI3 is the best target protein for bakuchiol Table 2 (Supplementary Tables 1–5).

**TABLE 2:** Interaction analysis and binding energies of the best target proteins.

S.No.	Protein/ligand	PDB ID	Binding energy	Interacting residues	
				H-bond(s)	Hydrophobic
1.	GNAI3/5468522	2ODE	−7.5 kcal/mol	A: Arg^158^(Bond length 2.3 Å)	A: Gln^44^, A: Gln^48^, A: Gly^51^, A: Val^53^, A: Thr^54^, A: Phe^55^, A: Gly^159^, A: Phe^162^, A: Gln^163^, A: Ile^166^
2.	PTGER3/5468522	6M9T	−6.9 kcal/mol	D: Asp^114^(Bond length 2.5 Å)	A: Arg^205^, A: Asp^237^, A: Glu^239^, B: Phe^109^, B: Asp^114^, B: Val^115^, B: Glu^116^, B: Phe^125^, B: Arg^128^, B: Glu^129^, B: Arg^132^, C: Arg^205^, C: Glu^236^, C: Asp^237^, C: Glu^239^, D: Phe^109^, D: Val^115^, D: Gln^116^, D: Phe^125^, D: Arg^128^, D: Arg^132^

### 3.3 Gene expression evaluation`

The present study aimed to explore the expression of GNAI3 and PTGER3 genes through the utilization of gene chip data in a TNM plot analyzer. Our findings, derived from extensive analysis, indicated that these genes were prominently expressed in lung tissue, with p-values of 2.21e-04 and 9.71e-16. Subsequently, we carried out a comparative analysis of these genes in normal, tumor, and metastatic tissues, which indicated a substantial decrease in their expression in metastatic tissues compared with that in tumor tissues. The p-values for these genes were 8.34e-04 and 4.51e-16, respectively (Supplementary Figure 7).

### 3.4 Biosynthesis of Bak-GNPs

In this study, Bakuchiol GNPs were synthesized using a biologically mediated method, with bakuchiol serving as both reducing and capping agent. Gold nanoparticles (GNPs) exhibit a characteristic reddish hue when suspended in aqueous solutions, which results from the excitation of surface plasmon vibrations (Wang et al., 2009). GNPs formation was confirmed through a color transformation from white to pink after approximately 24 h of incubation.

### 3.5 Characterization of synthesized nanoparticles

#### 3.5.1 UV-vis spectroscopy

**FIGURE 1:**
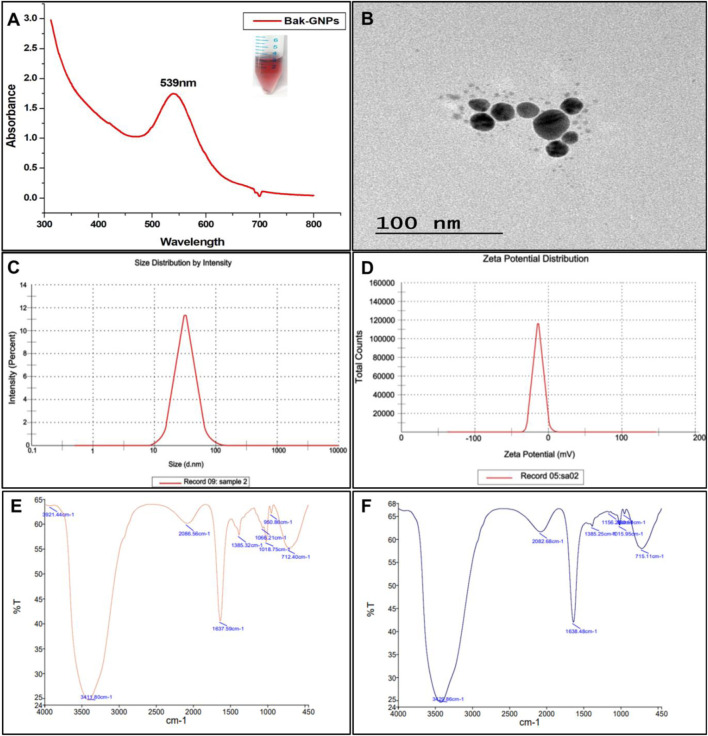
Characterization of Bak-GNPs: **(A)** UV-vis spectroscopy, **(B)** TEM, **(C)** DLS, **(D)** Zeta potential, **(E)** pure Bak FTIR, and **(F)** Bak-FTIR.

The initial exploration of the synthesized GNPs was conducted using UV-visible spectroscopy. It is worth noting that the intensity and width of surface plasmon resonance (SPR) peaks are contingent on factors such as the particle size, morphology, spatial arrangement, optical properties of the particles, and the surrounding medium. The bak-GNPs exhibited a prominent peak at 539 nm, confirming the formation of the GNPs Figure 1A (Mishra et al., 2023).

#### 3.5.2 FTIR (Fourier transform infrared spectroscopy)

FTIR measurements of pure Bakuchiol and Bak-GNPs were analyzed to identify the presence of various functional groups in pure bakuchiol (Figure 1E) and their role in stabilizing and capping GNPs (Figure 1F). For pure bakuchiol, prominent bands were observed at 3,411 cm^−1^ (O ─ H stretching), 1,642 cm^−1^ (C ═ C stretching), 1,385 cm^−1^ (C–H bending or C ─ F stretching), and 1,018 cm^−1^ (C–F stretching), suggesting the presence of alcohols, phenols, alkenes, and alkyl halides. These functional groups likely contributed to the stability/capping of the GNPs. A band at 950 cm^−1^ arising from the ═ C ─ H bending of the alkenes was observed. The band at 712 cm^−1^ was assigned to ═ C ─ H stretching or C ─ Cl stretching. Similar functional groups were identified in the Bak-GNPs, including O ─ H stretching (3,429 cm^−1^), C ═ C stretching (1,638 cm^−1^), ─ C ─ H bending/C ─ F stretching (1,385 cm^−1^), and C ─ F stretching/C ─ N stretching (1,156 cm^−1^). A band at 1,015 cm^−1^, arising from the C ─ F stretching of the alkenes, was observed. The bands at 950 cm^−1^ and 715 cm^−1^ were assigned to ═ C ─ H stretching and C ─ Cl stretching, respectively. These functional groups are indicative of the role of bakuchiol in the bioreduction of Au [III] and capping/stabilization of gold nanoparticles (Yuan et al., 2017).

#### 3.5.3 Dynamic light scattering (DLS) and Zeta potential

DLS analysis revealed a hydrodynamic diameter of ⁓55 d nm (Figure 1C) for the synthesized Bak-GNPs, providing insight into the physicochemical characteristics of the nanoparticles. The colloidal stability of the GNP solution was considered satisfactory when the magnitude of the zeta potential exceeded 30 mV, with higher absolute values indicating better stability (Berne and Pecora, 2000). The Zeta potential of Bak-GNPs was determined to be −12 mV, indicating a high level of stability Figure 1D. These findings suggest that Bak-GNPs exhibit excellent stability in aqueous suspensions owing to the electrostatic repulsive forces that prevent aggregation (Faried et al., 2016).

#### 3.5.4 Transmission electron microscopy (TEM)

TEM analysis showed the polydispersity and spherical shape of the Bak-GNPs, with an average particle size of approximately ⁓15 nm Figure 1B. Notably, the size observed via TEM was smaller than that obtained through DLS, as TEM measures the dry-state particle size, whereas DLS provides the hydrodynamic diameter in a hydrated state. This contrast highlights the influence of the solvent on the particle size measurement (Mollick et al., 2019).

### 3.6 Assessment of antimicrobial activity

The antibacterial effectiveness of a nanomaterial primarily relies on two key factors: the size of its particles and alterations made to its surface. Smaller particles exhibit heightened biological and chemical activities owing to their larger surface areas relative to their volumes. This increased surface area enhances the interaction between nanoparticles (NPs) and microorganisms, amplifying their efficacy (Brayner et al., 2006; Muthukumar et al., 2016). Bak-GNPs exhibit robust antibacterial activity against both Gram-positive and Gram-negative pathogenic bacterial strains.

**FIGURE 2:**
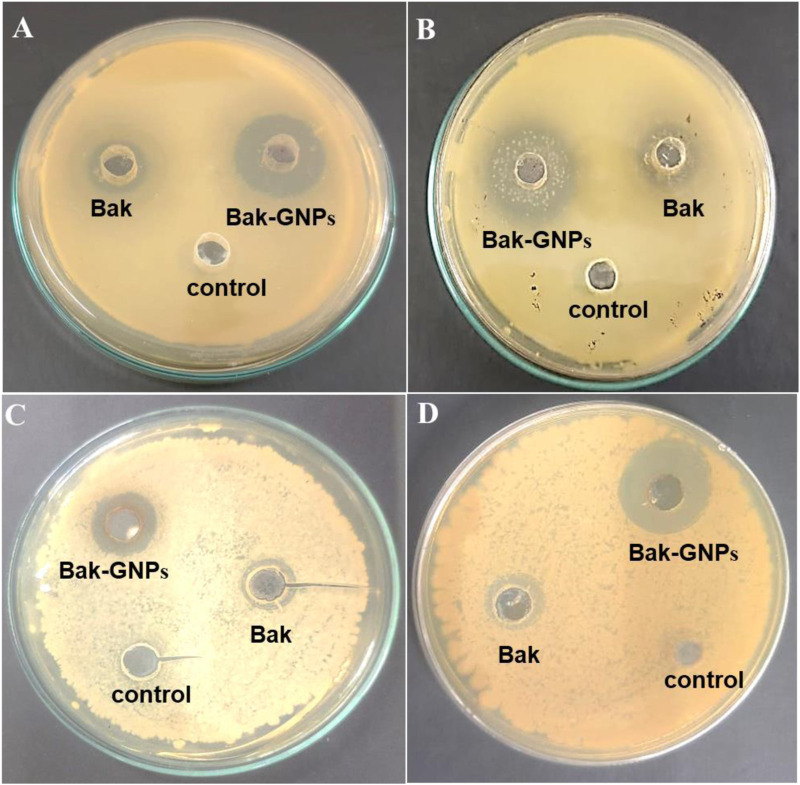
Antimicrobial activity of synthesized Bak-GNPs & pure Bak was determined using agar well diffusion method **(A)** *Escherichia coli* **(B)** *Klebsiella pneumoniae* **(C)** *Bacillus subtilis* **(D)** *Staphylococcus aureus*.

The synthesized Bak-GNPs, pure bakuchiol (Bak), and negative control (PBS) were added to the wells of MH agar plates. The experiments were conducted in triplicate and the agar plates were incubated overnight at 37°C. The diameter of the inhibitory zone was determined (Figure 2). In the following experiment, it was noted that pure bakuchiol (Bak) and Bak-GNPs diffused into the agar and strongly suppressed bacterial growth (Table 3).

**TABLE 3:** Zone of inhibition.

Bacterial strains	Zone of inhibition (mm)	
	Pure bakuchiol (Bak) (1 mg/mL) (mm)	Bak-GNPs (1 mg/mL) (mm)
*Bacillus subtilis*	15 ± 1.5	20 ± 1.5
*Klebsiella pneumoniae*	17 ± 2	24 ± 2
*Escherichia coli*	15 ± 1.5	23 ± 0.5
*Staphylococcus aureus*	16 ± 1.5	25 ± 1.7

**FIGURE 3:**
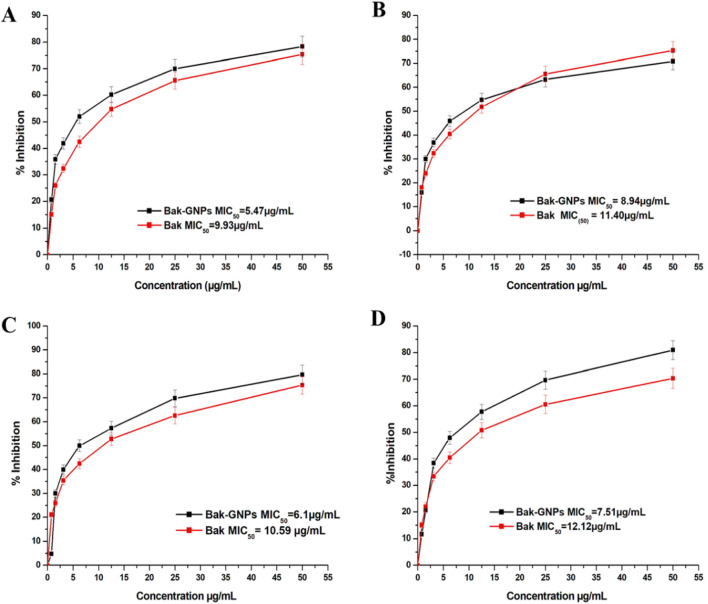
Minimum inhibitory concentration (MIC) of pure Bak and Bak-GNPs against **(A)** *Escherichia coli,* **(B)** *Klebsiella pneumoniae*, **(C)** *Bacillus subtilis*, and **(D)** *Staphylococcus aureus*. The data represent the mean ± standard error of three independent experiments.

The minimum inhibitory concentration (MIC_50_) values for pure bakuchiol and bak-GNPs against *Escherichia coli*, *Klebsiella pneumoniae*, *Bacillus subtilis*, and *Staphylococcus aureus* were quantified. Notably, Bak-GNPs displayed superior antibacterial efficacy, with lower MIC_50_ values than those of pure bakuchiol. Specifically, MIC_50_ values were determined as follows: 5.47 μg/mL (Bak-GNPs) vs. 9.93 μg/mL (Bak) for *Escherichia coli*; 8.94 μg/mL (Bak-GNPs) vs. 11.40 μg/mL (Bak) for *Klebsiella pneumoniae*; 6.11 μg/mL (Bak-GNPs) vs. 10.59 μg/mL (Bak) for *Bacillus subtilis*; and 7.51 μg/mL (Bak-GNPs) vs. 12.12 μg/mL (Bak) for *Staphylococcus aureus* (Figure 3). These results highlight the enhanced antibacterial potential of the Bak-GNPs (Khan et al., 2015). *Escherichia coli* and *Bacillus subtilis* exhibited superior antibacterial effects among the tested bacterial strains compared with the other two strains. Bak-GNPs demonstrated heightened effectiveness against bacteria at the lowest nanoparticle concentration. The variations in antibacterial performance observed across different bacterial species can be attributed to disparities in their cell wall compositions. According to a study by Kim et al. (2007), the interaction between bacteria and metallic GNPs involves binding to active sites on the cell membrane, thereby hindering cell cycle functions (Muthukumar et al., 2016). The antibacterial activity of Bak-GNPs demonstrated in our study offers a promising potential for the development of new antimicrobial agents. The efficacy of Bak-GNPs against various bacterial strains suggests that they could be utilized in designing novel antibacterial treatments, particularly for combating resistant strains that are not effectively addressed by current antibiotics. This aspect of our study could have significant implications for public health and the development of advanced therapeutic strategies.

### 3.7 Anticancer activity of Bak-GNPs

**FIGURE 4:**
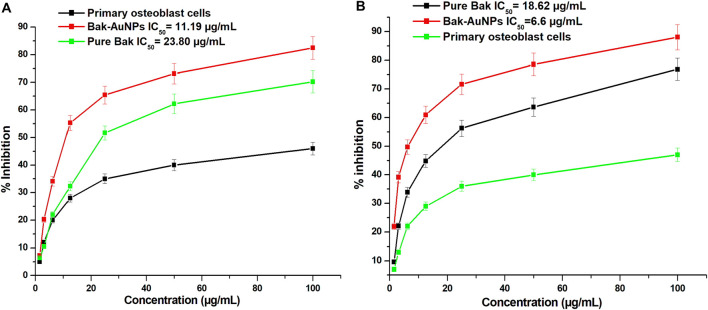
The cytotoxicity of gold nanoparticles produced from bakuchiol (Bak-GNPs) was assessed in two different cell lines: **(A)** A549 lung carcinoma cell line and **(B)** HepG2 liver cancer cells following treatment with varying doses of Bak-GNPs. The experiment was performed in triplicate and the data presented here represent the average values and standard errors.

This study investigated the anticancer potential of Bak-GNPs compared to that of pure Bak in lung (A549) and liver (HepG2) cancer cells. The results revealed that Bak-GNPs exhibited significantly greater anticancer activity than pure Bak. The inhibitory effect on cell growth increased with higher concentrations of Bak-GNPs and pure Bak. The IC_50_ values for Bak-GNPs and pure Bak on lung cancer cells were 11.19 and 23.80 μg/mL and liver cancer were 6.6 μg/mL and 18.62 μg/mL respectively, as shown in Figures 4A, B. This suggests that Bak-GNPs are more potent in inhibiting the viability of A549 and HepG2 cells.

### 3.8 Cell viability assessment of lung (A549) and liver (HepG2) cancer cell lines

To assess the effects of Bak-GNPs on A549 and HepG2 cells, various concentrations of Bak-GNPs were used. The viability of A549 cells declined rapidly within the concentration range of 1.56–100 μg/mL, ultimately resulting in an IC_50_ value of 11.19 μg/mL. The high selectivity of GNPs for cancer cells, as opposed to normal cells, highlights their potential as targeted therapeutic agents (Soliman et al., 2020). The effect of various concentrations of Bak-GNPs on the A549 lung carcinoma cell line and human primary osteoblast cells was evaluated using the MTT assay. The IC_50_ values of the synthesized Bak-GNPs was determined for lung cancer cells to be 11.19 μg/mL and for liver cancer cell to be 11.19 μg/mL. Consequently, a concentration of 6.6 μg/mL was chosen for subsequent experiments. Notably, no signs of toxicity were observed in human primary osteoblasts (Zhang et al., 2019).

### 3.9 Cytomorphological changes

**FIGURE 5:**
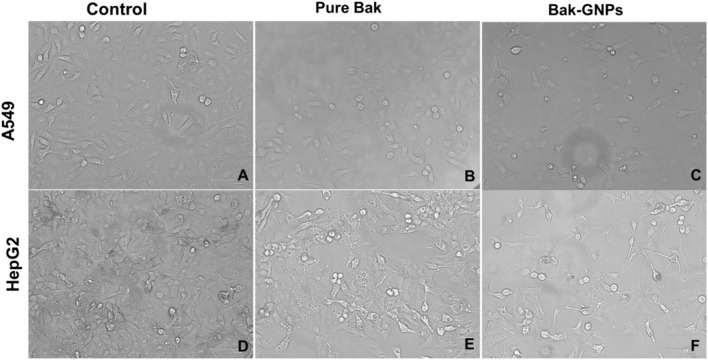
Cytomorphological images of A549 **(A-C)** and HepG2 **(D-F)** cells treated with IC_50_ (Bak-GNPs 11.19 μg/mL **(C)** and 6.6 μg/mL **(F)** and pure Bak 23.80 μg/mL **(B)** and 18.62 μg/mL **(E)**, respectively) concentration for 24 h analyzed by phase contrast microscopy. The images shown are representative of three independent experiments performed in triplicates.

Morphological changes in A549 and HepG2 Cells were examined after treatment with Bak-GNPs and Bak, respectively, at their respective IC_50_ concentrations. Control cells remained largely unchanged, whereas treated cells displayed detachment from the well surface, with some retaining intact plasma membranes, indicating the initiation of apoptosis. Pure Bak-treated cells exhibited signs of apoptosis but at significantly higher concentrations than those required for Bak-GNPs to induce apoptosis in lung cancer cells (Figure 5). This suggests that Bak-GNPs may induce apoptosis more effectively than pure Bak at lower concentrations.

### 3.10 Reactive oxygen species (ROS) generation

**FIGURE 6:**
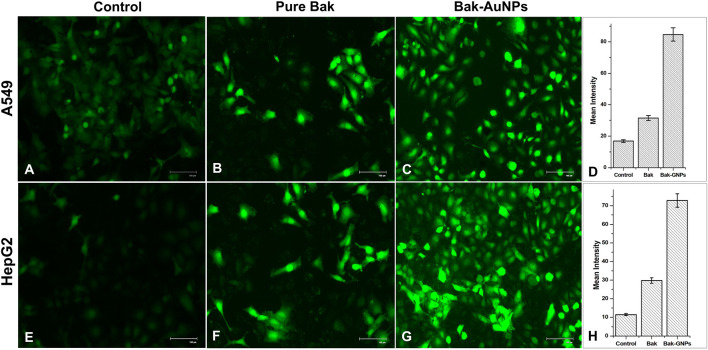
Qualitative evaluation of ROS in H_2_DCFDA-stained A549 **(A-C)** cell and HepG2 **(E-G)** cells treated at IC_50_ (Bak-GNPs 11.19 μg/mL **(C)** and 6.6 μg/mL **(G)** and pure Bak 23.80 μg/mL **(B)** and 18.62 μg/mL **(F)**, respectively) concentration for 24 h analyzed by fluorescence microscopy. The images shown are representative of three independent experiments. The fluorescence intensity of Bak and Bak-GNPs was evaluated in both A549 **(D)** and HepG2 **(H)** cells using IC_50_ values and compared to the values obtained from untreated cells. The mean signal intensities and standard deviations were determined for the treated cells.

ROS generation in A549 and HepG2 cells was assessed using DCFH-DA as the marker. Bak-GNP-treated A549 cells HepG2 Cells exhibited higher fluorescence levels than those in the control group, indicating increased ROS production. Pure Bak-treated cells also showed intense fluorescence; however, Bak-GNP-treated cells displayed even more intense fluorescence and disrupted morphological structures. This suggests that Bak-GNPs may induce oxidative stress and apoptosis through excessive generation of ROS, potentially contributing to their anticancer effects (Figure 6).

### 3.11 Nuclear morphology changes

**FIGURE 7:**
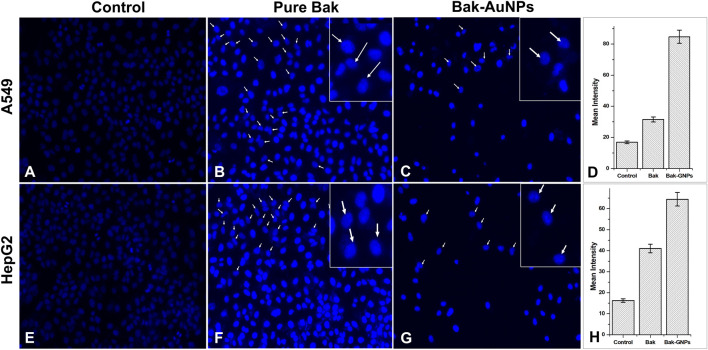
Nuclear morphology of DAPI stained nuclei of A549 **(A-C)** and HepG2 **(E-G)** cells treated at IC_50_ (Bak-GNPs 11.19 μg/mL **(C)** and 6.6 μg/mL **(G)** and pure Bak 23.80 μg/mL **(B)** and 18.62 μg/mL **(F)**, respectively) concentration for 24 h analyzed by fluorescence microscopy. The images shown are representative of three independent experiments. The fluorescence intensities of Bak and Bak-GNPs were evaluated in both A549 **(D)** and HepG2 **(H)** cells using IC50 values and then compared to those obtained from untreated cells. The mean signal intensities and standard deviations were determined for the treated cells.

To investigate the internalization of Bak-GNPs and their interaction with nuclear material, we used the fluorescent dye 4′, 6-diamidino-2-phenylindole (DAPI). A549 and HepG2 cancer cells were incubated for 48 h at 37°C, during which time they were exposed to Bak-GNPs at the IC_50_. The cells were stained with DAPI. Our microscopic analysis revealed striking differences in the nuclear morphology of A549 and HepG2 cells treated with Bak-GNPs compared with untreated cells. Notably, Bak-GNP-treated cells exhibited robust apoptotic effects, which were characterized by densely compacted chromatin and nuclei displaying consolidated dark blue fluorescence. This observation indicates significant changes in the structure and condensation of chromatin within the nucleus. The observed alterations in nuclear morphology and chromatin condensation suggested that Bak-GNPs may induce apoptosis in A549 cells. One possible mechanism for this apoptotic effect is an increase in cell membrane permeability (Iram et al., 2019). This may have allowed Bak-GNPs to enter the cells more effectively and interact with their nuclear material, ultimately leading to apoptosis. Such changes in membrane permeability can disrupt normal cell function and trigger programmed cell death (Figure 7).

To substantiate our findings, we conducted experiments in triplicates to ensure the reliability of our results. In summary, our investigation of the internalization of Bak-GNPs and their subsequent interaction with nuclear material, using DAPI staining, revealed substantial apoptotic effects in A549 and HepG2 cells. These effects are characterized by distinct changes in nuclear morphology, such as chromatin condensation, and are likely facilitated by an increase in cell membrane permeability (Syed et al., 2023).

Despite significant advances in cancer treatment, cancer management remains a formidable challenge. Although important milestones have been achieved in cancer therapy, the survival rate of patients with lung cancer remains disappointingly low. Patients with lung cancer are typically treated with cisplatin and other chemotherapeutic agents. However, these treatments resulted in a survival rate of approximately 20%–26% (Klastersky et al., 1989). Therefore, there is a pressing need to find an effective medication with minimal or no side effects for treating lung cancer. Nanomedicine represents a promising approach for cancer treatment (Peer et al., 2020). However, the conventional chemical synthesis of nanoparticles results in the production of toxic compounds, which can further exacerbate the condition of cancer patients. Consequently, in this study, we opted for a biological method to synthesize gold nanoparticles using bakuchiol, isolated from the seed of the Chinese tree *Psoralea corylifolia*. The cytotoxic effect of gold nanoparticles stems from their active physicochemical interactions with the functional groups of intracellular proteins, nitrogen bases, and phosphate groups within DNA. In a previous study by Sangiliyandi (2010) highlighted that nanoparticles with anticancer properties can inhibit the activities of abnormally expressed signaling proteins, such as Akt and Ras. Additionally, they can affect cytokine-based therapies, DNA- or protein-based vaccines targeting specific tumor markers, and tyrosine kinase inhibitors, all of which consistently demonstrate antitumor effects (Martins et al., 2010). This study observed anticancer activity, where the synthesized gold nanoparticles showed dose-dependent inhibition of lung and liver cells. However, some approved chemotherapeutic agents have side effects and are expensive. Thus, there is a critical need to develop alternative medicines to treat this serious disease. The discovery of synthesized gold nanoparticles aims to meet the demand for new treatments. Our findings on the anticancer properties of Bak-GNPs reveal their potential as effective agents for cancer therapy. The observed cytotoxicity against cancer cell lines indicated that Bak-GNPs may be used to enhance targeted cancer treatment approaches, potentially leading to new therapies with improved specificity and reduced side effects compared to conventional treatments.

## 4 Conclusion

This study presents a novel approach for the synthesis of GNPs using bakuchiol as the reducing and capping agent. The size and stability of GNPs were influenced by the bakuchiol concentration and incubation temperature, with optimal conditions yielding smaller-sized, highly uniform GNPs with superior zeta potential. The synthesized Bak-GNPs showed potent antibacterial activity against pathogenic strains and remarkable anticancer effects in A549 and HepG2 cells. These findings highlight the potential of Bak-GNPs as multi-faceted therapeutic agents. However, further research is required to comprehensively investigate the toxicity and underlying mechanisms of these effects. In summary, this study establishes a promising platform for the application of GNPs in diverse therapeutic approaches, paving the way for future investigations and potential clinical applications. This method of biosynthesis is straightforward, suitable for large-scale production, and is environmentally friendly.

## Data Availability

The datasets presented in this study can be found in online repositories. The names of the repository/repositories and accession number(s) can be found in the article/Supplementary Material.
