# Supplementary material for: Sustainable synthesis of bakuchiol-mediated gold nanoparticles for drug delivery against bacterial strains and tumor microenvironments, and its in silico target proteins identification
Source: Front Mol Biosci. 2024 Sep 25;11:1469107. doi: 10.3389/fmolb.2024.1469107 (PMC11462060; doi:10.3389/fmolb.2024.1469107)
Supplement: Supplementary file 1 [file DataSheet1.docx]

**Supplementary File**

**Sustainable synthesis of bakuchiol-mediated gold nanoparticles for drug delivery against bacterial strains and tumor microenvironments, and its *in silico* target proteins identification**

Pooja Mishra^1‡^, Tabrez Faruqui^1‡^, Sheeba Khanam^1^, Mohd. Khubaib^1^, Irfan Ahmad^2^, Mohd Saeed^3†^, and Salman Khan^1^*

1. Department of Biosciences, Integral University, Lucknow, Uttar Pradesh, India.
2. Department of Clinical Laboratory Sciences, College of Applied Medical Science, King Khalid University, Abha, Saudi Arabia.
3. Department of Biology, College of Sciences, University of Hail, Hail, Saudi Arabia.

**^†^**PRESENT ADDRESS

Mohd Saeed,

Centre for Global Health Research Saveetha Medical College, Chennai, India.

**^‡^**These authors have contributed equally to this work.

***Corresponding author mail**

**Salman Khan:** salmank@iul.ac.in


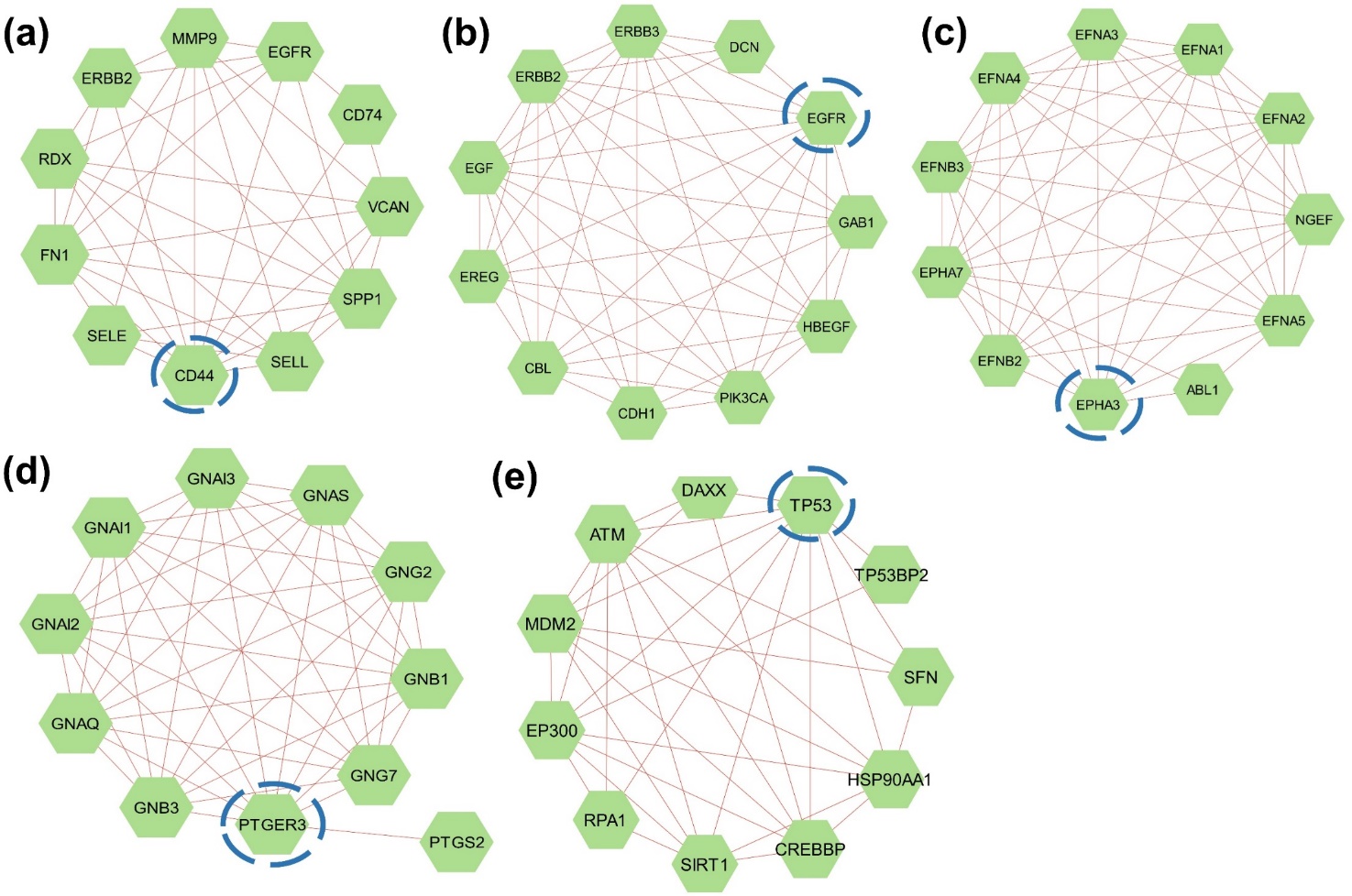


**S. Figure 1:** A network analysis of 5 selected target proteins **(a)** CD44, **(b)** EFGR, **(c)** EPHA3, **(d)** PTGER3, **(e)** TP53.


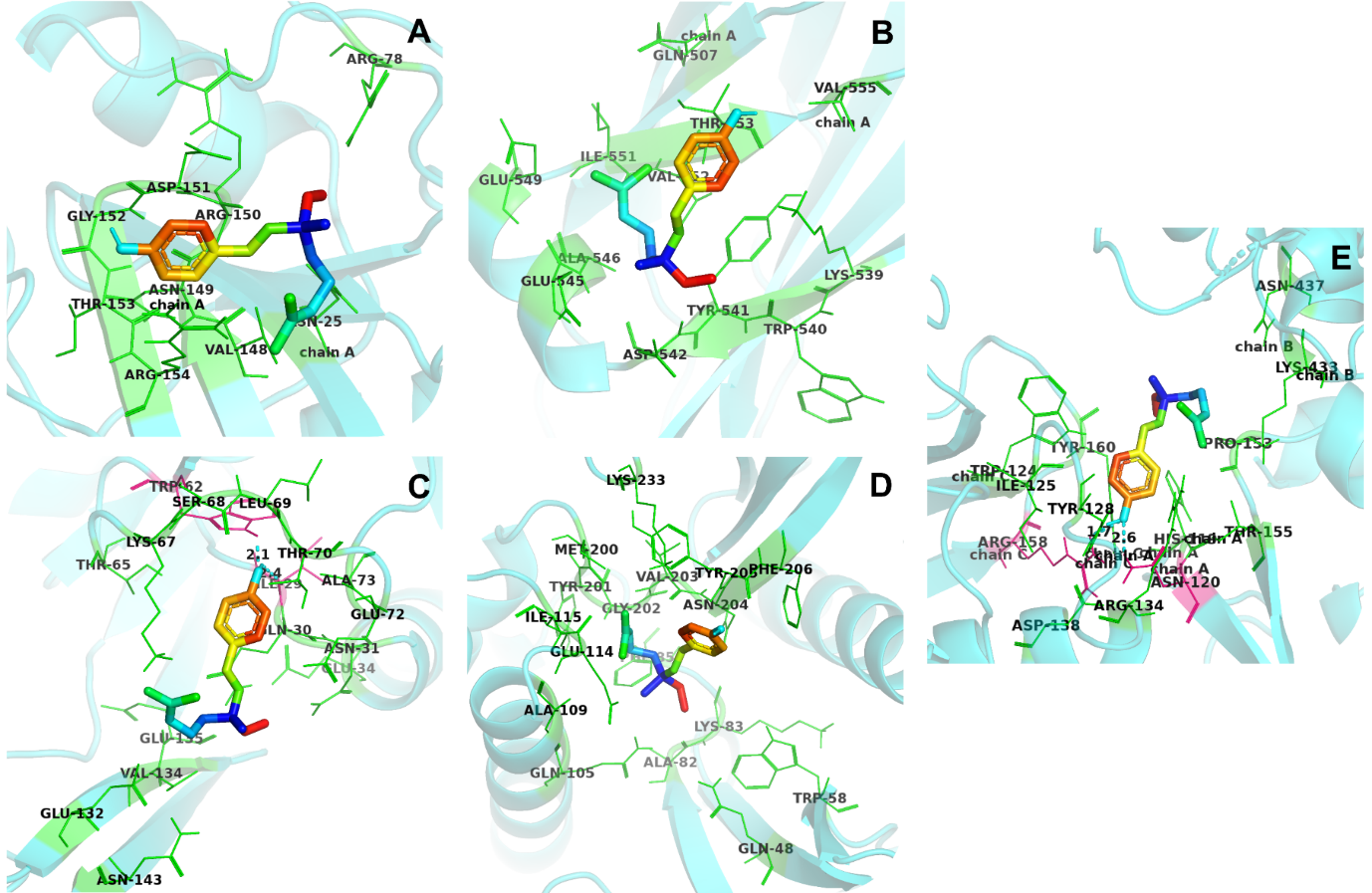


**S. Figure 2:** Interaction analysis of CD44 related 5 target proteins with bakuchiol.

**Note:** Hydrophobic residues are shown in green and magenta for hydrogen bonding.

**S. Table 1:** Binding energies and interacting residues by hydrogen bonds and hydrophobic interactions are shown in the table.

| **1. CD44** | | | | | |
| --- | --- | --- | --- | --- | --- |
| **S. No.** | **PDB ID/Compound** | **Binding energy** | **Number of H-bond** | **Hydrogen bond(s)** | **Hydrophobic interactions** |
| **1.** | **3X23/5468522** | − 6.0 | 0 | NA | A: Asn^25^, A: Arg^78^, A: Val^148^, A: Asn^149^, A: Arg^150^, A: Asp^151^, A: Gly^152^, A: Thr^153^, A: Arg^154^, |
| **2.** | **5TH6/5468522** | − 5.7 | 0 | NA | A: Gly^507^, A: Lys^539^, A: Trp^540^, A: Tyr^541^, A: Asp^542^, A: Glu^545^, A: Ala^546^, A: Glu^549^, A: Ile^551^, A: Val^552^, A: Thr^553^, A: Val^555^ |
| **3.** | **2HAZ/5468522** | − 5.5 | 2 | Trp^62^ (2.1 Å), Ile^29^ (2.4 Å) | Thr^65^, Lys^67^, Ser^68^, Leu^69^, Thr^70^, Ala^73^, Glu^72^, Gln^30^, Asn^31^, Glu^34^, Glu^132^, Val^134^, Glu^135^, Asn^143^ |
| **4.** | **3CFW/5468522** | − 5.2 | 0 | NA | Trp^58^, Gln^48^, Lys^83^, Ala^82^, Phe^85^, Gln^105^, Ala^109^, Ile^115^, Glu^114^, Met^200^, Tyr^201^, Gly^202^, Val^203^, Asn^204^, Tyr^205^, Phe^206^, Lys^233^ |
| **5.** | **1POZ/5468522** | − 4.0 | 2 | A: Asn^120^ (2.6 Å), C: Arg^158^ (1.7 Å) | A: His^119^, A: Pro^153^, A: Thr^156^, B: Lys^433^, B: Asn^437^, C: Ile^125^, C: Tyr^128^, C: Arg^134^, C: Asp^138^, C: Tyr^160^ |


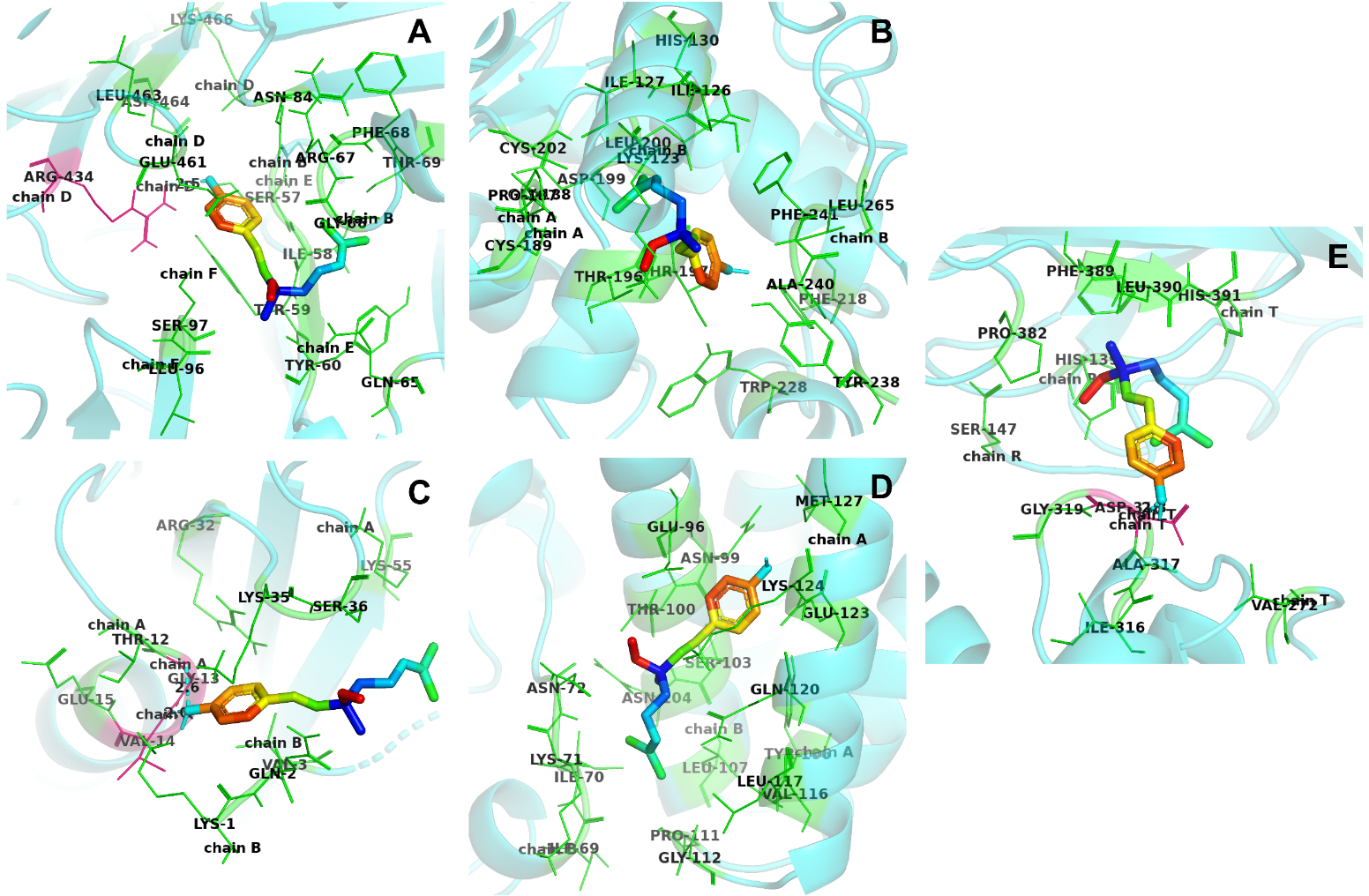


**S. Figure 3:** Interaction analysis of EGFR related 5 target proteins with bakuchiol.

**Note:** Hydrophobic residues are shown in green and magenta for hydrogen bonding.

**S. Table 2:** Binding energies and interacting residues by hydrogen bonds and hydrophobic interactions are shown in the table.

| **2. EGFR** | | | | | |
| --- | --- | --- | --- | --- | --- |
| **S. No.** | **PDB ID/Compound** | **Binding energy** | **Number of H-bond** | **Hydrogen bond(s)** | **Hydrophobic interactions** |
| **1.** | **7D85/5468522** | − 6.7 | 1 | D: Arg^434^ (2.5 Å) | B: Gly^66^, B: Arg^67^, B: Phe^68^, B: Thr^69^, B: Arg^67^, C: Glu^461^, C: Leu^463^, C: Asp^464^, C: Lys^466^, C: Ser^57^, C: Ile^58^, C: Tyr^59^, C: Tyr^60^, C: Gln^65^, F: Leu^96^, F: Ser^97^ |
| **2.** | **3VRG/5468522** | − 6.4 | 0 | NA | A: Pro^187^, A: Gly^188^, A: Cys^189^, B: Lys^123^, B: Ile^126^, B: Ile^127^, B: His^130^, B: Thr^196^, B: Thr^197^, B: Asp^199^, B: Leu^200^, B: Cys^202^, B: Phe^218^, B: Trp^228^, B: Tyr^238^, B: Ala^240^, B: Phe^241^, B: Leu^265^ |
| **3.** | **4GSY/5468522** | − 6.1 | 2 | A: Gln^13^ (2.6 Å), Val^14^ (2.0 Å) | A: Thr^11^, A: Glu^15^, A: Arg^32^, A: Lys^35^, A: Ser^36^, A: Lys55, B: Lys^1^, B: Gln^2^, B: Val^3^ |
| **4.** | **7LEM/5468522** | − 6.1 | 0 | NA | A: Tyr^106^, A: Phe^111^, A: Gly^112^, A: Val^116^, A: Lys^117^, A: Gln^120^, A: Glu^123^, A: Lys^124^, A: Met^127^, B: Ile^69^, B: Ile^70^, B: Lys^71^, B: Asn^72^, B: Glu^96^, B: Asn^99^, B: Thr^100^, B: Ser^103^, B: Asn^104^, B: Leu^107^ |
| **5.** | **1XDT/5468522** | − 4.8 | 1 | T: Asp^318^ (2.3 Å) | R: His^139^, R: Ser^147^, T: Val^272^, T: Ile^316^, T: Ala^317^, T: Asp^318^, T: Gly^319^, T: Pro^382^, T: Phe^389^, T: Leu^390^, T: His^391^ |


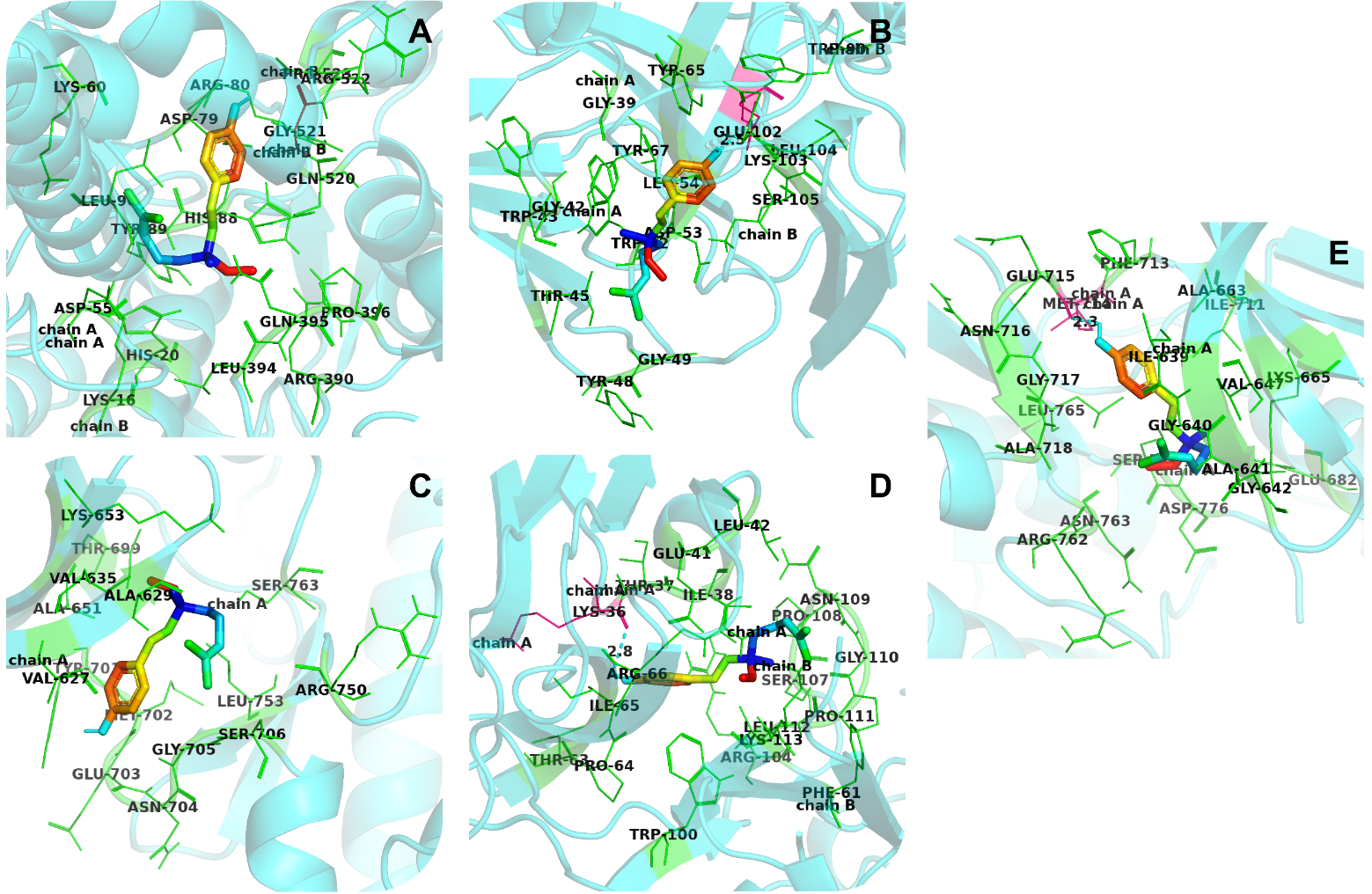


**S. Figure 4:** Interaction analysis of EPHA3 related 5 target proteins with bakuchiol.

**Note:** Hydrophobic residues are shown in green and magenta for hydrogen bonding.

**S. Table 3:** Binding energies and interacting residues by hydrogen bonds and hydrophobic interactions are shown in the table.

| **3. EPHA3** | | | | | |
| --- | --- | --- | --- | --- | --- |
| **S. No.** | **PDB ID/Compound** | **Binding energy** | **Number of H-bond** | **Hydrogen bond(s)** | **Hydrophobic interactions** |
| 1. | **4I0P/5468522** | − 6.5 |  | NA | A: Asp^55^; B: Lys^16^, B: His^20^, B: Lys^60^, B: Asp^79^, B: Arg^80^, B: His^88^, B: Tyr^89^, B: Lys^90^, B: Arg^390^, B: Leu^394^, B: Glu^395^, B: Pro^396^, B: Glu^520^, B: Gly^521^, B: Arg^522^, B: Pro^523^ |
| 2. | **7ECC/5468522** | − 6.0 | 1 | A: Gln^56^ (2.5 Å) | A: Gly^39^, A: Gly^42^, A: Trp^43^, A: Thr^45^, A: Tyr^48^, A: Gly^49^, A: Trp^52^, A: Asp^53^, A: Leu^54^, A: Tyr^65^, A: Tyr^67^; B: Thr^90^, B: Glu^102^, B: Lys^103^, B: Leu^104^, B: Ser^105^ |
| 3. | **4G2F/5468522** | − 5.8 |  | NA | Val^627^, Ala^629^, Val^635^, Ala^651^, Lys^653^, Thr^699^, Tyr^701^, Met^702^, Glu^703^, Asn^704^, Gly^705^, Ser^706^, Arg^750^, Leu^753^, Ser^763^ |
| 4. | **2W03/5468522** | − 5.8 | 1 | A: Lys^36^ | A: Thr^37^, A: Ile^38^, A: Glu^41^, A: Leu^42^, A: Thr^63^, A: Pro^64^, A: Ile^165^, A: Arg^66^, B: Phe^61^, B: Trp^100^, B: Arg^101^, B: Ser^107^, B: Pro^108^, B: Asn^109^, B: Gly^110^, B: Pro^111^, B: Leu^112^, B: Lys^113^ |
| 5. | **3CZU/5468522** | − 5.6 | 1 | A: Met^714^ (2.3 Å) | A: Ile^639^, A: Gly^640^, A: Ala^641^, A: Gly^642^, A: Val^647^, A: Ala^663^, A: Lys^665^, A: Glu^682^, A: Ile^711^, A: Phe^713^, A: Glu^715^, A: Asn^716^, A: Gly^717^, A: Ala^718^, A: Arg^762^, A: Asn^763^, A: Leu^765^, A: Ser^775^, A: Asp^176^ |


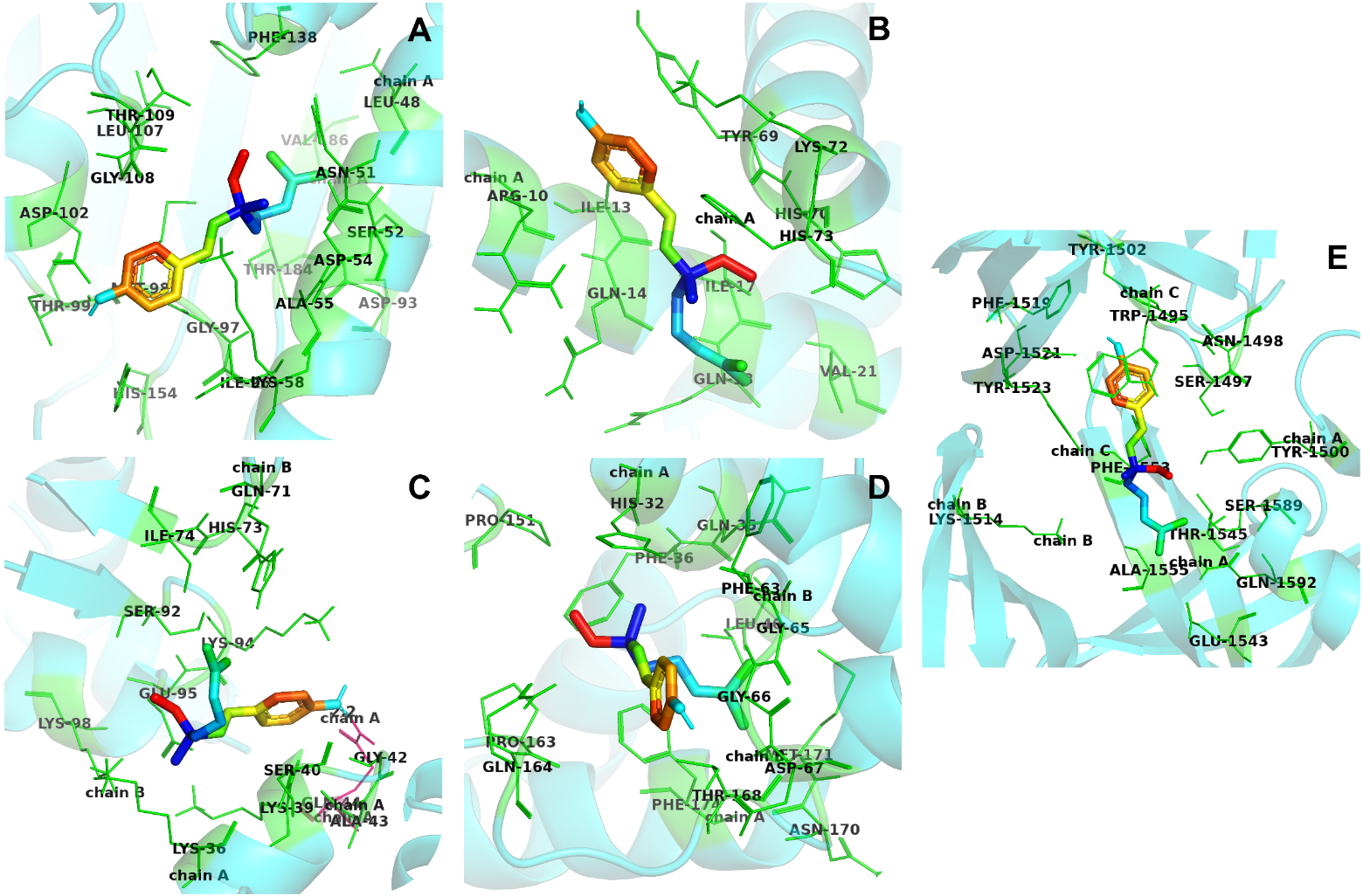


**S. Figure 5:** Interaction analysis of PTGER3 related 5 target proteins with bakuchiol.

**Note:** Hydrophobic residues are shown in green and magenta for hydrogen bonding.

**S. Table 4:** Binding energies and interacting residues by hydrogen bonds and hydrophobic interactions are shown in the table.

| **4. PTGER3** | | | | | |
| --- | --- | --- | --- | --- | --- |
| **S. No.** | **PDB ID/Compound** | **Binding energy** | **Number of H-bond** | **Hydrogen bond(s)** | **Hydrophobic interactions** |
| 1. | **2ODE/5468522** | − 7.5 | 1 | A: Arg^158^ (2.3 Å) | A: Gln^44^, A: Gln^48^, A: Gly^51^, A: Val^53^, A: Thr^54^, A: Phe^55^, A: Gly^159^, A: Phe^162^, A: Gln^163^, A: Ile^166^ |
| 2. | **6M9T/5468522** | − 6.0 | 1 | D: Asp^114^ (2.5 Å) | A: Arg^205^, A: Asp^237^, A: Glu^239^, B: Phe^109^, B: Asp^114^, B: Val^115^, B: Glu^116^, B: Phe^125^, B: Arg^128^, B: Glu^129^, B: Arg^132^, C: Arg^205^, C: Glu^236^, C: Asp^237^, C: Glu^239^, D: Phe^109^, D: Val^115^, D: Gln^116^, D: Phe^125^, D: Arg^128^, D: Arg^132^ |
| 3. | **6CRK/5468522** | − 5.8 | 0 | NA | A: Lys^210^, A: His^213^, B: Tyr^59^, B: Ala^60^, B: Met^61^, B: Met^101^, B: Thr^102^, B: Cys^103^, B: Ser^147^, B: Cys^148^, B: Arg^150^, B: Met^188^, B: Ser^189^, B: Leu^190^, B: Ser^191^, B: Leu^192^, B: Asn^230^, B: Ile^232^, B: Cys^233^, B: Thr^274^, B: Ser^275^, B: Val^276^, B: Ser^316^, B: Cys^317^, B: Leu^318^ |
| 4. | **7YR7/5468522** | − 5.8 | 0 | NA | A: Lys^83^, A: Lys^85^, A: Phe^88^, A: Arg^155^, A: Ala^158^, A: Ile^159^, A: Pro^162^, A: Tyr^165^, A: Leu^256^, A: Trp^273^ |
| 5. | **2BCJ/5468522** | − 4.4 | 1 | I: Cys^215^ (2.0 Å) | I: Asp^26^, I: Gly^27^, I: Ala^30^, I: Ala^31^, I: Lys^35^, I: His^214^, I: Cys^215^, I: Phe^216^, I: Glu^217^, I: Gly^218^; T: Leu^55^, T: Ala^56^, T: Lys^57^, T: Gln^75^, T: Asp^76^, T: Trp^99^ |


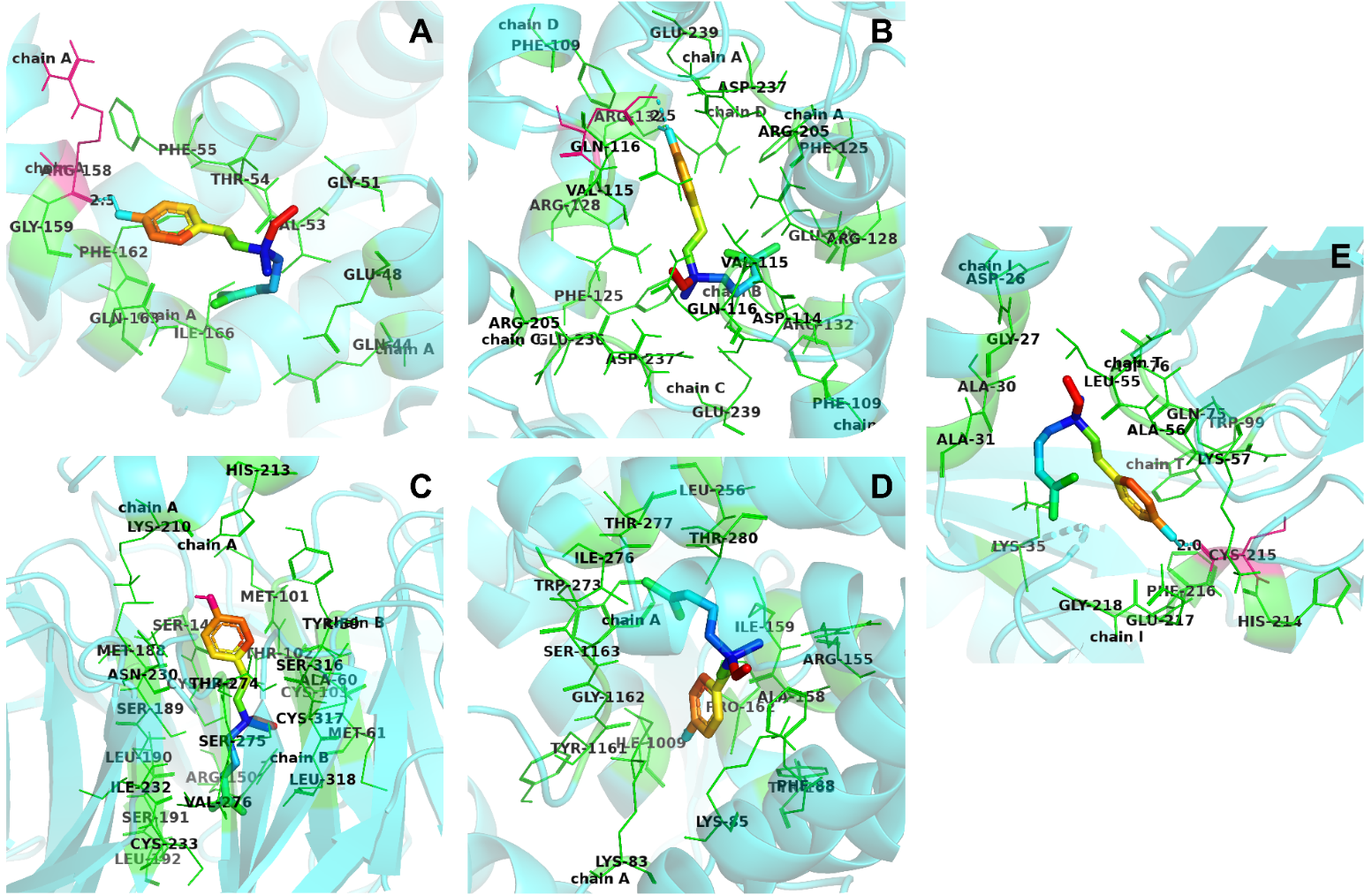


**S. Figure 6:** Interaction analysis of TP53 related 5 target proteins with bakuchiol.

**Note:** Hydrophobic residues are shown in green and magenta for hydrogen bonding.

**S. Table 5:** Binding energies and interacting residues by hydrogen bonds and hydrophobic interactions are shown in the table.

| **5. TP53** | | | | | |
| --- | --- | --- | --- | --- | --- |
| **S. No.** | **PDB ID/Compound** | **Binding energy** | **Number of H-bond** | **Hydrogen bond(s)** | **Hydrophobic interactions** |
| 1. | **7S1C/5468522** | − 6.8 | 0 | A: Arg^158^ (2.5 Å) | A: Leu^48^, A: Asn^51^, A: Ser^52^, A: Asp^54^, A: Ala^55^, A: Lys^58^, A: Asp^93^, A: Ile^96^, A: Gly^97^, A: Met^98^, A: Thr^99^, A: Asp^102^, A: Leu^107^, A: Gly^108^, A: Thr^109^, A: Phe^138^, A: His^154^, A: Thr^184^, A: Val^186^ |
| 2. | **1UYL/5468522** | − 6.3 | 0 | NA | Arg^10^, Ile^13^, Gln^14^, Gln^18^, Ile^17,^ Val^21^, Tyr^69^, Lys^72^, His^70^, His^73^ |
| 3. | **3T92/5468522** | − 5.9 | 1 | A: Gln^44^ (2.2 Å) | A: Lys^36^, A: Lys^39^, A: Ser^40^, A: Gly^42^, A: Ala^43^; B: Gln^71^, B: His^73^, B: Ile^74^, B: Ser^92^, B: Lys^94^, B: Glu^95^, B: Lys^98^ |
| 4. | **8SWJ/5468522** | − 5.8 | 0 | NA | A: His^32^, A: Gln^35^, A: Phe^36^, A: Leu^40^, A: Pro^151^, A: Pro^163^, A: Gln^164^, A: Thr^168^, A: Asn^170^, A: Met^171^, A: Phe^174^, B: Phe^63^, B: Gly^65^, B: Gly^66^, B: Asp^67^ |
| 5. | **6Q9L/5468522** | − 4.9 | 0 | NA | A: Tyr^1500^, A: Glu^1543^, A: Phe^1553^, A: Ala^1555^, A: Ser^1589^, A: Gln^1592^, A: Lys^1514^, C: Trp^1494^, C: Ser^1497^, C: Asn^1498^, C: Tyr^1502^, C: Phe^1519^, C: Asp^1521^, C: Tyr^1523^ |

**S. Table 5** Binding energies of 25 target proteins.

| **S. No.** | **Protein Name/**  **Gene name** | **PDB ID** | **Binding energy** |
| --- | --- | --- | --- |
| **1. CD44** | | | |
| 1 | RDX | 3X23 | **− 6.0** |
| 2 | MMP9 | 5TH6 | **− 5.7** |
| 3 | FN-1 | 2HAZ | **− 5.5** |
| 4 | SELL | 3CFW | **− 5.2** |
| 5 | CD44 | 1POZ | **− 4.0** |
| **2. EGFR** | | | |
| 6 | ERBB3 | 7D85 | **− 6.7** |
| 7 | CBL | 3VRQ | **− 6.4** |
| 8 | GAB1 | 4QSY | **− 6.1** |
| 9 | EREG | 7LEM | **− 6.1** |
| 10 | HVEGF | 1XDT | **− 4.8** |
| **3. EPHA3** | | | |
| 11 | EFNA5 | 4LOP | **− 6.5** |
| 12 | EPHA7 | 7ECC | **− 6.0** |
| 13 | EHPA3 | 4G2F | **− 5.8** |
| 14 | EFNAZ | 2WO3 | **− 5.8** |
| 15 | EFNA1 | 3CZU | **− 5.6** |
| **4. PTGER3** | | | |
| 16 | GNAI3 | 2ODE | **− 7.5** |
| 17 | PTGER3 | 6M9T | **− 6.9** |
| 18 | GNAI1 | 6CRK | **− 5.8** |
| 19 | GNAI2 | 7YK7 | **− 5.8** |
| 20 | GNAQ | 2BCJ | **− 4.4** |
| **5. TP53** | | | |
| 21 | ATM | 7S1C | **− 6.8** |
| 22 | HSP90AA1 | 1UYL | **− 6.3** |
| 23 | EP300 | 3T92 | **− 5.9** |
| 24 | TP53 | 8SWJ | **− 5.8** |
| 25 | MDM2 | 6Q9L | **− 4.9** |





**S. Figure 7:** Gene expression of GNAI3 and PTGER3 in lung and liver tissues.
